# Factors associated with prefectural level physical activity in Japan: An ecological study

**DOI:** 10.1002/jgf2.707

**Published:** 2024-06-19

**Authors:** Noboru Horinouchi, Yuki Kataoka, Kyoko Yamamoto, Seiji Shiota, Eishi Miyazaki

**Affiliations:** ^1^ Department of General Medicine Oita University Faculty of Medicine Oita Japan; ^2^ Department of Internal Medicine Kyoto Min‐Iren Asukai Hospital Kyoto Japan; ^3^ Section of Clinical Epidemiology, Department of Community Medicine, Graduate School of Medicine Kyoto University Kyoto Japan; ^4^ Department of Healthcare Epidemiology Kyoto University Graduate School of Medicine/Public Health Kyoto Japan; ^5^ Scientific Research Works Peer Support Group (SRWS‐PSG) Osaka Japan

**Keywords:** health insurance claim, National Database, older adults

## Abstract

**Background:**

Physical activity inequalities are an important global concern; nonetheless, few studies have comprehensively examined the association between physical activity inequalities and related factors. We aimed to explore factors associated with regional inequalities in physical activity in Japan.

**Methods:**

We investigated the relationship of individual, psychological/behavioral, social, and built‐environmental factors with the proportion of individuals with exercise habits across Japanese prefectures. We sourced data from open databases, including the Japanese National Health Insurance Claims and Nationwide Screening Program Database. We defined exercise habits as engaging in at least 30 min of moderate exercise at least twice a week. We employed multiple regression analysis to identify factors associated with prefectural level physical activity.

**Results:**

The median proportion of individuals with exercise habits was 27% (interquartile range, 25–29). Higher frequency of exercise habits was associated with higher frequency of history of exercise (coefficients, 0.60; 95% confidence interval, 0.41–0.80) and lower proportion of female gender (coefficients, −1.74; 95% confidence interval, −2.80 to −0.69).

**Conclusions:**

Exercise history and female gender may be related to prefectural level physical activity in Japan. Physical activity interventions should be promoted among women without exercise history.

## Graphical abstract

We comprehensively explore the association of psychological, behavioral, built‐environmental, and socio‐environmental factors with regional inequality in Japan. We showed in this study that female gender and prior exercise habits might be associated with prefectural level physical activity in Japan.

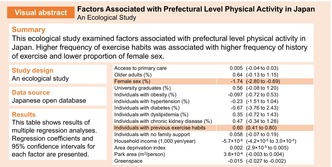

## 1 BACKGROUND

Physical inactivity, which has grown into a global concern, influences various diseases and mortality rates.^1^, ^2^ Although the implementation of various policies to promote physical activity in the past decade has resulted in slow progress in this domain, gaps in progress remain, leading to global inequalities in physical activity.^3^, ^4^ In a previous study, the proportion of the population with sufficient physical activity and the Gini coefficient have been calculated to examine inequalities in physical activity.^5^ Considering the negative effects of physical inactivity, reducing these inequalities is important for promoting public health.^3^, ^4^, ^5^, ^6^

Various factors have been associated with individual physical activity,^7^, ^8^ including psychological (e.g., self‐efficacy), behavioral **(**e.g., previous physical activity**)**, built‐environmental (e.g., land use mix), and socioenvironmental factors (e.g., support from family). However, whether inequalities in these factors are associated with regional level physical activity remains unclear. Although examined at the regional level,^9^, ^10^ the association between architectural environmental factors and differences in physical activity at the prefectural level throughout Japan remains unclear. A recent cross‐sectional study found that individual physical activity is associated with socioeconomic inequality, including the size of the city of residence.^11^ Pabayo et al.^12^ showed that state‐level income inequality in the United States is associated with physical activity among women. Nonetheless, studies comprehensively examining the association between physical activity inequalities across regions and physical activity‐related factors, including psychological/behavioral, built‐environment, and socioenvironmental factors, are sparse.

This ecological study aimed to explore factors associating prefectural level physical activity in Japan by analyzing the relationship between various factors and the regional distribution of individuals with exercise habits.

## 2 METHODS

### 2.1 Study design

This cross‐sectional ecological study was conducted within the framework of the Japanese Nationwide Screening Program and analyzed data from 47 Japanese prefectures. As an ecological study using an open database, it was exempted from ethical approval. The study was conducted and reported following the Strengthening the Reporting of Observational Studies in Epidemiology guidelines (Table S1).^13^

### 2.2 Data sources

We sourced age, gender, exercise habits, and health status data from the open database of “National Database of Health Insurance Claims and Specific Health Checkups of Japan (NDB).”^14^ This open database contains the following data (medical, dental, and dispensing practice fees, specific health checkup questions and answers, and laboratory data) by 47 prefectures in Japan in a non‐personally identifiable format. Specific Health Checkups targets individuals aged 40–74 years who are covered by the Japanese National Health Insurance system. The NDB open database provides information on age, gender, body mass index, use of antihypertensive, hypoglycemic, and dyslipidemia medications, exercise habits, and renal function. All data, except those on exercise habits, were retrieved from the latest available open data, dated from 2019.^14^

Data on the area and number of clinics in each prefecture were obtained from the Japanese Medical Association database.^15^ Data on education, household composition, and employment status were acquired from the 2020 Japanese population census.^16^ Data on household income were obtained from the 2019 national survey of family income and expenditure.^17^ Data on the total area of park and greenspace in each prefecture were extracted from the Japanese Social and Population Statistics system.^18^ This database contains data on various aspects of the Japanese society, including social welfare.

### 2.3 Candidate exposures

Individual, psychological/behavioral, social, and built‐environmental factors have been associated with physical activity.^6^, ^7^ Among these, we considered as candidate exposures the factors with available data in the open databases. For each prefecture, we calculated the proportion of individuals receiving the Nationwide Screening Program who met each criterion from the NDB open database.

Among individual factors, the age and gender variables were defined as the proportion of individuals who were 65 years of age or older and female, respectively. The health status variable was the proportion of individuals with obesity and individuals with hypertension, diabetes, dyslipidemia, and chronic kidney disease. Obesity was defined as a body mass index of ≥25.0 kg/m^2^. Hypertension, diabetes, and dyslipidemia were defined as a history of taking drugs for each condition. Chronic kidney disease was defined as an estimated glomerular filtration rate of <60 mL/min/1.73 m^2^. Among behavioral factors, the variable of interest was exercise history, defined as the proportion of individuals with exercise habits in 2018.^19^

Among social factors, the number of clinics per 100 km^2^ in each prefecture was considered as an indicator of access to primary care. The proportion of university graduates in each prefecture was used as the education variable. The social support variable was the proportion of individuals with no family support, which was calculated by dividing the number of single‐person households by the total number of households in each prefecture. The neighborhood deprivation variable was defined as the area deprivation index,^20^ which is obtained by combining the following indicators: proportion of older adult couple households, older adult single‐person households, mother and child households, households living in rented accommodation, gray‐collar workers, agricultural and fishery workers, blue‐collar workers, and unemployment rate. The income variable was defined as the average annual income per household in each prefecture. We included environmental factors available at the 47‐prefecture level in Japan from open data as candidate factors among those shown to be associated with physical activity at the individual level. Built‐environmental factors included the total areas of park developed for exercise and greenspace in each prefecture.

### 2.4 Study outcome

The study outcome was the proportion of individuals with exercise habits in each prefecture. Subjects in the Japanese Nationwide Screening Program are asked whether they perform moderate exercise for at least 30 min at least twice a week. We calculated the proportion of program subjects who answered “yes” to this question in each prefecture using data from the 2019 NDB open database.^14^ We considered this proportion to be the proportion of individuals with exercise habits.

### 2.5 Statistical analyses

We inspected all variable distributions visually. Normally distributed outcome and candidate variables were summarized as means with standard deviations, and those with skewed distribution as medians with interquartile ranges (IRQ). We conducted multiple regression analyses to examine the association between the proportion of individuals with exercise habits in each prefecture and candidate variables. The following variables that were not normally distributed were log‐transformed: access to primary care, individuals with exercise habits, individuals with no family support, and green space. The distribution of the proportion of individuals with exercise habits in each Japanese prefecture was mapped based on log‐transformed proportions. We used the variance inflation factor to check the multicollinearity among variables. We determined that if the variance inflation factor (VIF) was less than 10, the multicollinearity of the variable was acceptable. This threshold is arbitrary and a higher VIF means greater multicollinearity. All data analyses and map creation were performed using R (version 4.0.5).

## 3 RESULTS

**FIGURE 1:**
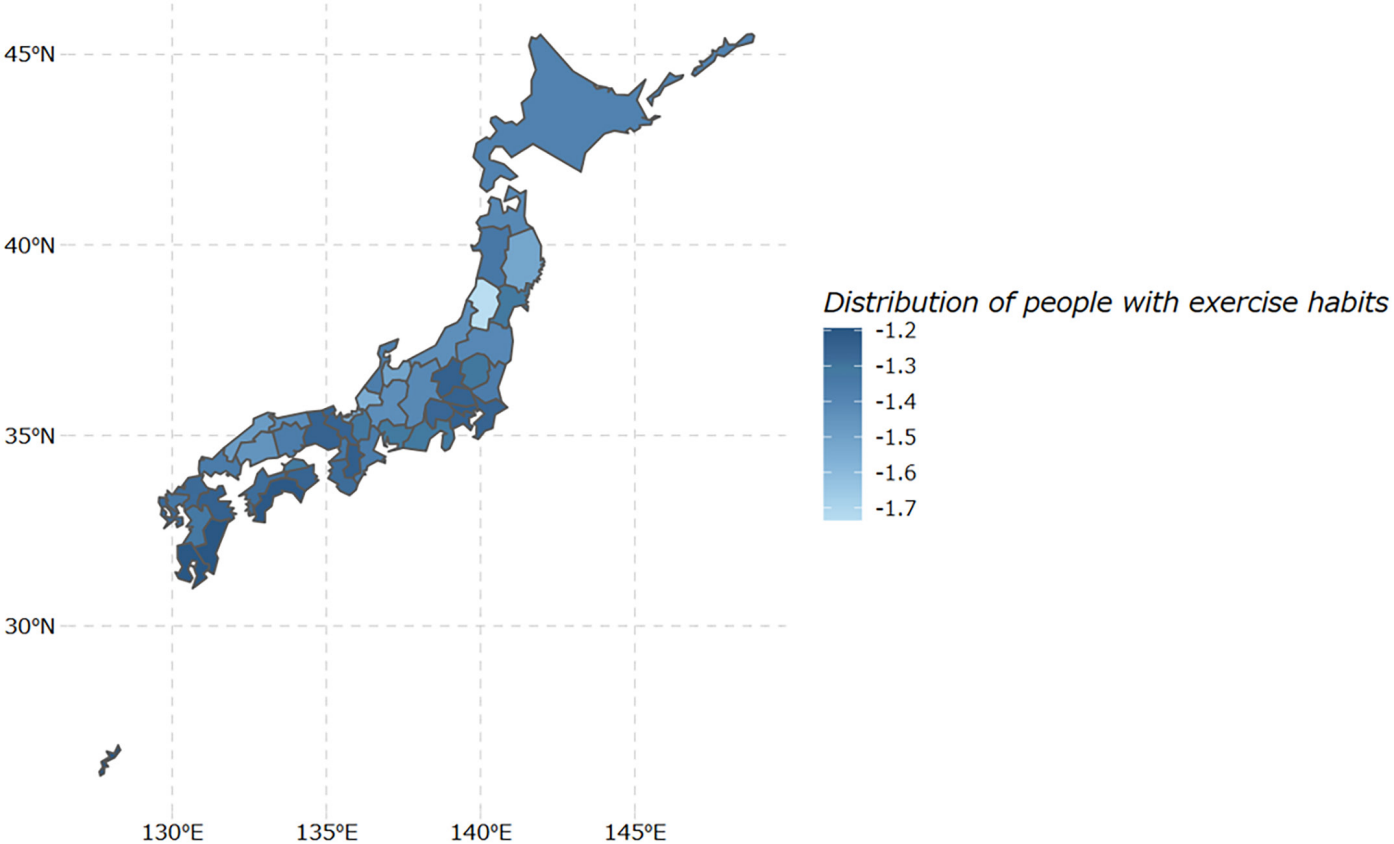
The distribution of the proportion of individuals with exercise habits was log‐transformed and mapped. On the map, the darker the blue color, the higher the proportion of individuals with exercise habits.

Table 1 shows the distribution of the candidate exposures. The median value for access to primary care was 57.9 (IQR, 38.5–81.1) per 100 km^2^. The median proportion of individuals with exercise habits was 27% (IQR, 25–29; range, 18–30). Figure 1 shows the distribution of the proportion of individuals with exercise habits in each prefecture in Japan. The darker the blue color, the higher the proportion of individuals with exercise habits. Figure 1 suggests that the proportion of individuals with exercise habits is high in the part of Kanto and Kinki regions, and in the southern part of Kyushu and Shikoku regions. The Gini coefficient^21^ calculated for the proportion of individuals with exercise habits was 0.054.

**TABLE 1:** Distribution of the candidate exposures in 47 prefectures.

Exposures	
Access to primary care (*n*/100 km^2^), median (IQR)	57.9 (38.5–81.1)
Older adults (%), mean (SD)	26 (4)
Female gender (%), mean (SD)	35 (2)
University graduates (%), mean (SD)	17 (4)
Individuals with obesity (%), mean (SD)	29 (3)
Individuals with hypertension (%), mean (SD)	22 (3)
Individuals with diabetes (%), mean (SD)	6 (1)
Individuals with dyslipidemia (%), mean (SD)	15 (1)
Individuals with chronic kidney disease (%), mean (SD)	10 (1)
Individuals with previous exercise habits (%), median (IQR)	26 (25–28)
Individuals with no family support (%), median (IQR)	34 (32–37)
Household income (1000 yen/year), mean (SD)	5403.0 (532.3)
Area deprivation index, mean (SD)	74.9 (9.1)
Park area (m^2^/person), mean (SD)	11.9 (4.2)
Greenspace (ha), median (IQR)	165.2 (95.5–333.3)
Outcome	
Individuals with exercise habits (%), median (IQR)	27 (25–29)

Abbreviations: IQR, interquartile range; SD, standard deviation.

The results of the single and multiple regression analyses are shown in Table 2. In the multiple regression analyses, higher frequency of exercise history and lower proportion of female gender was associated with higher proportion of individuals with exercise habits. The confidence intervals for the coefficients of the area deprivation index and greenspace did not include a zero value; however, the coefficients were near zero in magnitude. The *p*‐value for *F*‐statistics in the multiple regression analysis model was less than 0.001 and multiple *R*‐squared was 0.95.

**TABLE 2:** Results of the single and multiple regression analyses of the candidate exposures.

Exposures	Single regression		Multiple regression	
	Coefficients	95% CI	Coefficients	95% CI
Access to primary care	0.05	0.02–0.09	−0.005	−0.04 to 0.03
Older adults (%)	0.15	−0.71 to 1.02	0.64	−0.13 to 1.15
Female gender (%)	−4.00	−5.43 to −2.56	−1.74	−2.80 to −0.69
University graduates (%)	0.85	0.05–1.65	0.56	−0.08 to 1.20
Individuals with obesity (%)	0.48	−0.66 to 1.62	−0.097	−0.72 to 0.53
Individuals with hypertension (%)	−0.34	−1.60 to 0.92	−0.23	−1.51 to 1.04
Individuals with diabetes (%)	−0.83	−5.86 to 4.19	−0.67	−3.76 to 2.43
Individuals with dyslipidemia (%)	−2.15	−4.69 to 0.39	0.35	−0.72 to 1.43
Individuals with chronic kidney disease (%)	1.68	−0.86 to 4.22	0.47	−0.34 to 1.28
Individuals with previous exercise habits	0.91	0.82–1.01	0.60	0.41–0.80
Individuals with no family support	0.49	0.25–0.74	0.058	−0.07 to 0.19
Household income (1000 yen/year)	−6.88 × 10^−5^	−1.3 × 10^−4^ to −1.21 × 10^−5^	−5.7 × 10^−6^	−4.2 × 10^−5^ to 3.0 × 10^−5^
Area deprivation index	0.01	0.003–0.009	0.002	2.9 × 10^−5^ to 0.005
Park area (m^2^/person)	−0.01	−0.02 to −0.003	3.8 × 10^−4^	−0.003 to 0.004
Greenspace	0.01	−0.017 to 0.037	−0.015	−0.027 to −0.002

*Note*: The following exposures were log‐transformed: access to primary care, individuals with previous exercise habits, individuals with no family support, and greenspace.

Abbreviation: CI, confidence interval.

Based on the regional distribution of prefecture level physical activity shown in Figure 1, we performed an ad hoc multiple regression analysis by adding annual sunlight duration (hour/year) in each prefecture in 2019 as a weather factor.^22^ The ad hoc multiple regression analysis did not show association between annual sunlight duration and prefecture level physical activity (coefficient, −1.66 × 10–5; 95%CI, −1.08 × 10^−4^ to 7.73 × 10^−5^). Table 3 shows proportions of females and individuals with previous exercise habits in each region of Japan. Table 3 suggests that the Kanto and Kinki regions had fewer females, and individuals with previous exercise habits were more common in the Kanto and Kyushu/Okinawa regions than in others.

**TABLE 3:** Proportions of females and individuals with previous exercise habits in each region of Japan.

Exposures	Hokkaido/Tohoku	Kanto	Chubu	Kinki	Chugoku/Shikoku	Kyushu/Okinawa
Female gender (%)	37.1 (35.9–37.2)	32.7 (32.5–33.2)	35.9 (34.7–36.0)	34.3 (34.2–34.6)	35.6 (34.2–36.7)	35.6 (34.7–36.3)
Individuals with previous exercise habits (%)	24.6 (22.9–25.6)	28.8 (28.1–29.1)	24.8 (23.6–26.7)	27.3 (26.2–28.3)	25.5 (23.0–25.5)	28.5 (27.3–29.8)

*Note*: Each exposure is summarized by median and interquartile range.

In the main analysis, the VIF of access to primary care and proportion of hypertension exceeded 10. We also performed multiple regression analyses with candidate variables other than hypertension as an ad‐hoc sensitivity analysis (Table 4). In this model, the regression coefficients were almost like those in the main analysis, and the VIF for each variable was less than 10.

**TABLE 4:** Results of the multiple regression analyses of the candidate exposures without hypertension.

Exposures	Multiple regression	
	Coefficients	95% CI
Access to primary care	−0.12	−0.04 to 0.02
Older adults (%)	0.60	0.15 to 1.04
Female gender (%)	−1.76	−2.80 to −0.72
University graduates (%)	0.62	0.07 to 1.17
Individuals with obesity (%)	−0.16	−0.66 to 0.34
Individuals with diabetes (%)	−0.83	−3.57 to 1.65
Individuals with dyslipidemia (%)	−0.96	−0.69 to 1.24
Individuals with chronic kidney disease (%)	0.45	−0.34 to 1.24
Individuals with previous exercise habits	0.60	0.42–0.79
Individuals with no family support	0.06	−0.06 to 0.19
Household income (1000 yen/year)	−5.22 × 10^−6^	−4.1 × 10^−5^ to 3.0 × 10^−5^
Area deprivation index	0.002	7.8 × 10^−5^ to 0.005
Park area (m^2^/person)	3.0 × 10^−4^	−0.003 to 0.004
Greenspace	−0.014	−0.026 to −0.002

*Note*: The following exposures were log‐transformed: access to primary care, individuals with previous exercise habits, individuals with no family support, and greenspace.

Abbreviation: CI, confidence interval.

## 4 DISCUSSION

This ecological study showed that, in Japan, exercise history may be associated with high, while female gender with low prefectural level physical activity. Although the multiple regression analysis revealed a statistical association between the area deprivation index, greenspace availability, and exercise habits, the magnitude of the association was minimal and lacked clinical significance.

Our findings align with those of previous studies on the correlation between gender and exercise history,^8^, ^23^, ^24^ suggesting the necessity of endorsing exercise implementation among women and individuals without a history of physical activity to mitigate disparities in physical activity levels. Goal setting, self‐monitoring, and supportive counseling have proven to be effective behavior modification techniques to encourage physical activity.^25^ Prioritizing these interventions, particularly among women without a history of exercise, might contribute to reducing inequalities in physical activity.

In our study, the area deprivation index was not associated with physical activity at the prefectural level in Japan. Previous studies have shown mixed results. In Australia, area‐level characteristics were related to smoking, obesity, and quality of life, but not physical activity.^26^ In South Korea, area level socioeconomic status correlated with drinking, smoking, and physical activity.^27^ The definitions of area‐level indicators and physical activity in these studies differed from ours, which may explain the different results. For example, in the Korean study defined physical activity as walking at least 30 min a day, for five or more days in a week.^27^ Further studies using consistent criteria are needed to examine the association between physical habits and indicators of socioeconomic status at the regional level.

Regarding green space availability, our results were not consistent with previously reported findings. The Korean Green Belt Policy, a strategy aimed at protecting and managing the green spaces surrounding urban areas, was shown to be associated with a decrease in the prevalence of obesity in Korea.^28^ This finding was attributed to the fact that the availability of green spaces may have increased the levels of residents' leisure time physical activity. Our study analyzed data on exercise habits but did not obtain data on the type of physical activity, which may be the reason for the discrepancy in the results on green space availability. Our findings imply the need for further research on the relationship between the built environment, including green space availability, and the type of physical activity in Japan.

The following factors were not associated with physical activity at the prefectural level in the current study: access to primary care, proportion of the following factors (older adults, university graduates, obesity, hypertension, diabetes, dyslipidemia, chronic kidney disease, and family support), park area, and household income. Previous studies have shown that various factors are associated with physical activity at the individual level.^6^, ^7^, ^8^ This hypothetical exploratory study comprehensively examined the association between these factors and physical activity at the prefectural level. Future confirmatory studies are needed to examine the association between each factor and prefectural level physical activity individually.

This study has several limitations that should be considered when interpreting the results. First, as it was an ecological study, caution should be exercised when interpreting the results because of the possibility of ecological fallacies. For example, the results of this study should not be interpreted to imply that individual‐level physical activity is higher among residents living in areas with a higher proportion of female gender compared to residents living in areas with a lower proportion of female gender. Second, because of the cross‐sectional design, a causal relationship between each exposure and physical activity cannot be established. Third, the open data utilized in this study were not all obtained in the same year. Interpretation of the results should be approached with caution, particularly for the employment status data from fiscal year 2020, as they may have been impacted by COVID‐19. Finally, the respondents to the Japanese Nationwide Screening Program were skewed toward higher age groups in each prefecture (data not shown), thereby restricting the generalizability of the findings. Longitudinal studies covering a broader range of generations are needed to clarify the relationships between each of the factors analyzed in this study and exercise habits, as well as their underlying mechanisms. While these weaknesses should be considered, this study also has its strengths. The main strength is the use of national data to investigate prefecture‐level differences in exercise habits and associated factors in Japan. A confirmatory study using individual‐level data rather than prefecture‐level data is needed to test the hypotheses made in this study.

In conclusion, our ecological study suggests a relationship between the proportion of female gender and prior exercise habits, and prefectural level physical activity among Japanese adults. These results underscore the importance of promoting physical activity interventions in regions with a high proportion of women without a history of exercise. Further research is warranted to validate the efficacy of such interventions.

## AUTHOR CONTRIBUTIONS

All authors contributed to the study conception and design. Data collection and analysis were performed by Noboru Horinouchi. The first draft of the manuscript was written by Noboru Horinouchi and Yuki Kataoka, and all authors critically revised the manuscript for important intellectual content. All authors have read and approved the final version of the manuscript.

## CONFLICT OF INTEREST STATEMENT

Authors declare no conflicts of interests for this article.

## ETHICS APPROVAL STATEMENT

As an ecological study using open database, it was exempted from ethical approval.

## PATIENT CONSENT STATEMENT

None.

## CLINICAL TRIAL REGISTRATION

None.

## Supporting information


Table S1.

## Data Availability

This study was conducted using an open database.
